# Implementation of Microfluidic Sandwich ELISA for Superior Detection of Plant Pathogens

**DOI:** 10.1371/journal.pone.0083231

**Published:** 2013-12-23

**Authors:** Numrin Thaitrong, Ratthaphol Charlermroj, Orawan Himananto, Channarong Seepiban, Nitsara Karoonuthaisiri

**Affiliations:** National Center for Genetic Engineering and Biotechnology, National Science and Technology Development Agency, Klong Luang, Pathum Thani, Thailand; University of Houston, United States of America

## Abstract

Rapid and economical screening of plant pathogens is a high-priority need in the seed industry. Crop quality control and disease surveillance demand early and accurate detection in addition to robustness, scalability, and cost efficiency typically required for selective breeding and certification programs. Compared to conventional bench-top detection techniques routinely employed, a microfluidic-based approach offers unique benefits to address these needs simultaneously. To our knowledge, this work reports the first attempt to perform microfluidic sandwich ELISA for *Acidovorax citrulli* (Ac), watermelon silver mottle virus (WSMoV), and melon yellow spot virus (MYSV) screening. The immunoassay occurs on the surface of a reaction chamber represented by a microfluidic channel. The capillary force within the microchannel draws a reagent into the reaction chamber as well as facilitates assay incubation. Because the underlying pad automatically absorbs excess fluid, the only operation required is sequential loading of buffers/reagents. Buffer selection, antibody concentrations, and sample loading scheme were optimized for each pathogen. Assay optimization reveals that the 20-folds lower sample volume demanded by the microchannel structure outweighs the 2- to 4-folds higher antibody concentrations required, resulting in overall 5–10 folds of reagent savings. In addition to cutting the assay time by more than 50%, the new platform offers 65% cost savings from less reagent consumption and labor cost. Our study also shows 12.5-, 2-, and 4-fold improvement in assay sensitivity for Ac, WSMoV, and MYSV, respectively. Practical feasibility is demonstrated using 19 real plant samples. Given a standard 96-well plate format, the developed assay is compatible with commercial fluorescent plate readers and readily amendable to robotic liquid handling systems for completely hand-free assay automation.

## Introduction

Seed trade is a fast growing industry of more than 10% average annual growth rate since 2005 [1]. Thailand, in particular, has become one of the largest seed producers and exporters in Asia-Pacific with over 100 million US dollars in annual revenue [2]. Faced with increasing demand, the industry is in need of rapid and reliable methods to screen seedborne pathogens that, if present, can pose serious threats to not only the business worldwide but also the global food supply [3]. Each year, crop diseases account for several millions to billions of dollars losses around the world [4]. These pathogens ranging from bacteria [5], viruses [6], [7], fungi [8], and parasites [9] reduce both quality and quantity of agricultural products as well as result in trade bans on exporters. Aside from disease surveillance and management, disease epidemiological studies and selective breeding programs can also benefit from accurate and cost-effective screening methods.

Although various diagnostic methods have been applied for detecting seed and plant pathogens, each approach has different advantages and shortcomings. The most popular molecular-based methods, such as polymerase chain reaction (PCR) and probe-based tests [10], provide extremely specific and sensitive results, but the techniques require sterile conditions and complex nucleic acid extraction and purification [11]. Newer technique such as loop-mediated isothermal amplification [12], [13] though can suffer from similar drawbacks, has started to gain interest due to its frequently improved assay performance and simpler instrumentation over traditional PCR. The technique has been used to detect Plum Pox virus [14], bacteria in potatoes [15], and fungi in bananas [16], to name a few. In contrast, insensitive microscopic inspection is straightforward and rapid, but it does require highly experienced pathologists. Finally, extensively adopted immunoassays [12] such as enzyme-linked immunosorbent assays (ELISA) offer a simpler operation and a high level of sensitivity. The method, nevertheless, requires a large amount of reagents, several time-consuming incubating and washing steps, rendering it inefficient for an industrial-scale adoption. Given these limitations, many recent efforts in bioanalytical research, thus, have shifted to microfluidic technology for improved assay performance, throughput, cost, speed, and ease-of-use [17].

Microfluidic systems or micro total analysis systems (μTAS) offer several desirable advantages such as greater sensitivity, faster turnaround time, and lower sample consumption, owing to unique properties of miniaturization such as small volume, large surface-to-volume ratio, short diffusion distance, laminar flow, and high surface tension [18], [19]. Highly flexible platform design also allows for integration, automation, and portability. Finally, massively parallel systems can be inexpensively fabricated with high level of precision and consistency by highly streamlined microfabrication techniques routinely used in semiconductor and integrated circuit industries.

Though staggering progress in miniaturization technology has been made in the field of biotechnology during the past decades [20], [21], applications of microfluidic systems in agricultural applications are still very limited. A few instances of these developments include DNA microarray on an open-channel microfluidic chip for detecting *Boytrytis cinerea, Botrytis sqamosa*, and *Didymella bryoniae*−plant fungal pathogens responsible for greenhouse crop diseases [22]. Unlike stationary microarray, probes and sample were flown through the orthogonal channel design and accelerated hybridization occurred at the intersections. A microfluidic disk containing a double spiral structure accommodating 384 × 384 hybridization assays was developed for fungal plant testing's [23]. Fluid delivery was controlled by the centrifugal pumping, eliminating the need for auxiliary pumps and valves.

In this work, we describe implementation of the next generation commercial microfluidic-based microplate to perform sandwich ELISA for screening plant pathogens commonly found in cucurbits. To our knowledge, this miniaturized system has only been instrumental in human cytokine biomarker (IL4) detection [24] and cell signaling pathway study [25]. This study, thus, presents the first demonstration of this miniaturized sandwich ELISA technology for detecting seedborne pathogens: *Acidovorax avenae* subsp. *citrulli* (Ac) causing fruit blotch in watermelon [26], watermelon silver mottle virus (WSMoV), and melon yellow spot virus (MYSV). Taking advantages of low volume (5 µL in a microfluidic channel vs. 100 µL in a traditional ELISA well), fast reaction kinetics (10 min vs. 1 hr), and simple operating procedure (no reagent removal steps) offered by the capillary channels within the microplate [27], the developed microfluidic-based assay could provide a new and improved benchmark for rapid and accurate screenings of agricultural products. Here, we optimized and characterized analytical performance of the microfluidic system in comparison with the traditional ELISA method. Technological feasibility and practicality of the system were also investigated using real plant samples. This validated system serves as a model diagnostic platform that is readily compatible with robotic automation, making it suitable for large-scale, routine screening.

## Materials and Methods

### Antibody preparation

All antibodies used in this study were summarized in Table 1. All antibodies, except MPC, were obtained from the Monoclonal Antibody (MAb) Production Laboratory, BIOTEC, Thailand [28], [29]. MPC was purchased from the Department of Plant Pathology, Faculty of Agriculture, Kasetsart University (Kamphaeng Saen Campus) in Thailand. Secondary polyclonal antibody (PAb), MPC and MYSV6, were conjugated with alkaline phosphatase (AP) using an ALK Phos conjugation kit (AbD Serotec, LNK012AP, United Kingdom). Briefly, 10 µL of LL-modifier was added to 100 µg of an antibody suspended in 100 µL filtered phosphate buffered saline (PBS, pH 7.4 containing 1 mM KH_2_PO_4_, 0.15 mM Na_2_HPO_4_, and 3 mM NaCl). The reaction mixture was incubated overnight at room temperature (RT). Additiion of a 10-µL quencher stopped the reaction, and the conjugated antibodies were stored at 4°C.

**Table 1:** Plant pathogen detection panel.

Pathogen	Capture MAb/Source	Detection PAb/Source
*Acidovorax Citrulli* (Ac)	11E5/mouse	AP-MPC/rabbit
*watermelon silver mottle virus* (WSMoV)	2D6/mouse	AP-MYSV6/rabbit
*melon yellow spot virus (MYSV)*	5E7/mouse	AP-MYSV6/rabbit

“AP” denotes alkaline phosphate conjugate.

### Sample preparation

*Acidovorax avenae* subsp. *citrulli* (Ac) was inoculated in nutrient broth (Difco™ laboratory, #234000) for 16 hr at 200 rpm, 30°C. The cells were resuspended in PBS, and the concentrations were determined from OD_600_ measurements (Cintra spectrometer 404, GBC Scientific Equipment, USA). The corresponding colony-forming unit (CFU) was calculated using a conversion factor of 3 × 10^9^ CFU/mL per 1 OD_600_ unit, as previously determined by the plate count method.

Nucleocapsid coat proteins (NPs) of watermelon silver mottle virus (WSMoV) and melon yellow spot virus (MYSV) were purchased from the Plant Research Pathology Laboratory, Agricultural Biotechnology Research Unit, BIOTEC (Kamphaeng Saen Campus, Thailand). Standard antigens were prepared by PCR amplification of nucleocapsid protein genes from tospovirus-infected plants using gene specific primers (GenBank accession numbers: AY514625 and AY574574 for WSMoV and MYSV, respectively) [30]. Each PCR product was cloned into pQE80L expression vector (Qiagen) with 6×His tag at the N-terminus, which was then transformed into *E. coli* (DH5α). After protein expression was induced by isopropyl-1-thio-β-D galactoside (IPTG, 1 mM), the 6×His-tagged protein was purified using a Ni-NTA agarose resin column under denaturing condition. The NPs were determined to be ∼30 kDa [31].

The standard antigens were diluted in 1% skim milk for traditional ELISA experiments, and in OptiBlock (Siloam Biosciences, Cincinnati, OH) for microfluidic-based experiments. To prepare plant extracts, 0.5 g of dried plant leaves were suspended in 20 mL of the corresponding diluents and manually ground. The solution was centrifuged at 13,000 g for 10 min at 4°C and the supernatant was collected. Spiked plant extracts were prepared by diluting antigens of known concentrations in the disease-free watermelon extract. For real sample tests, plant specimens were collected from a watermelon field (Amphur Songpeenong, Suphan Buri province, Thailand, collection date: 25 Dec 2012). No specific permits or permissions were required for the described locations and activities. The location is not privately owned or protected in any way. The field studies did not involve endangered or protected species. Upon collection, fresh leaves were stored in small sample collection tubes at −80°C. At the time of experiments, 20 mL of diluent was added to the whole sample before grinding. Dried leaves stored at −20°C were processed in a similar manner as previously described.

### Traditional ELISA assays

**Figure 1:**
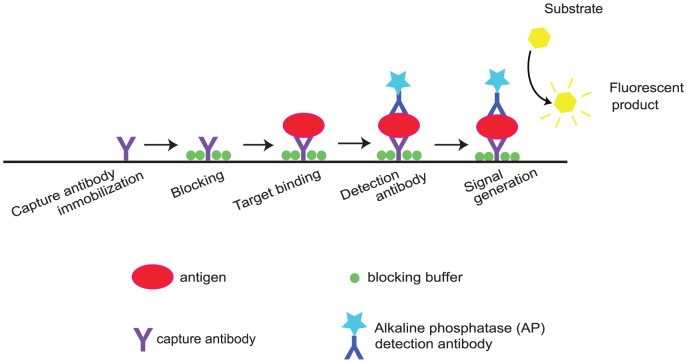
Schematic workflow depicting sequential molecular binding events of the sandwich ELISA. Within each reaction chamber, the capture antibody is adsorbed on the reactive surface followed by surface passivation by a blocking buffer. Upon target binding to the capture antibody, alkaline phosphates (AP)-tagged detection antibody specific to the antigen is added. Addition of fluorescent substrate (PNPP or *p*-nitrophenyl phosphate for the traditional well format, and Attophos for the micofluidic format) activated by AP generates detectable fluorescent signal, indicating successful binding events.

The assay panel summarized in Table 1 was first verified by traditional sandwich ELISA on a 96-well plate format (Costar, 3590) based on a previously reported protocol [28]. The workflow of the assay was depicted in Figure 1. Briefly, the plate was coated with 100 µL of 2.5 µg/mL capture MAb diluted in a sodium carbonate-bicarbonate buffer, pH 9.6. After an overnight incubation at 4°C, the plate was brought to RT and washed with 300 µL of PBST buffer (PBS with 0.05% Tween 20) three times prior to being blocked with 100 µL of 3% skim milk in PBST for 1 hr. Following another washing step, antigens (100 µL of 10^7^ CFU/mL of Ac, 5 µg/mL of WSMoV-NP and 5 µg/mL of MYSV-NP) prepared in 1% skim milk in PBST were added to the wells corresponding to their specific MAb (Table 1), and incubated for 1 hr at RT. Following the third washing was a 1-hr incubation in 100 µL of paired detection PAb at 0.5 µg/mL, 1 µg/mL, 2 µg/mL, and 4 µg/mL. The plate was washed again followed by adding 200 µL of Alkaline Phosphate Yellow (PNPP or *p*-nitrophenyl phosphate, Invitrogen #00-2212) and incubating for 15 min. The absorbance measurements were recorded at 405 nm (Multiskan FC microplate photometer, Thermo Scientific). Optimal antibodies concentrations, which gave the highest signal to noise (S/N) ratios, were selected for assay transfer and baseline sensitivity. The S/N ratio was measured by taking an average of the signal readouts divided by an average of the noise readouts from negative controls. Limits of detection for each pathogen were determined using S/N = 2 as a cutoff value [32].

### Assay transfer to the microfluidic system

All experiments were performed on the 96-well format microfluidic cartridges (Optimiser™, Siloam) following the workflow in Figure 1. Similar to the traditional ELISA, the microfluidic channel was first coated with MAb, followed by a washing and a blocking step. An antigen of interest was then sandwiched between bound MAb and AP-tagged PAb. For the channel volume of 4.5 µL, only 5 µL of reagents was needed for each 10-min incubation step [27], with the exceptions of final washing (30 µL, 10 min), sample incubation (5 µL, 20 min), and fluorescent substrate incubation (10 µL, 15 min) steps. After washing out the unbound AP-tagged PAb, the fluorescent substrate PNPP was added and the fluorescent signal was read at λ_ex_ = 440 nm, λ_em_ = 560 nm (Synergy Mx, BioTek).

#### Selection of coating buffers

To select an optimal binding buffer for each pathogen, 2.5 µg/mL of the capture MAb (11E5, 2D6, and 5E7) were prepared in binding buffers OptiBind A-L (Siloam, #OMR-50). The same set of binding buffers containing no antibodies was included as a negative control. Following the initial incubation, the channels were washed and blocked with the OptiWash and OptiBlock solutions (Siloam), respectively, according to the manufacturer's protocol. Immobilized MAbs were reacted with AP-conjugated goat anti-mouse IgG (Sigma #10765: diluted 1∶3000 in OptiBlock) for 20 min before being washed twice. In the final step, 10 µL of Attophos fluorescent substrate (Promega, #S1001) was added and incubated for 15 min before recording fluorescent signals.

#### Selection of blocking buffers

For each pathogen, the channels were coated with 10 µg/mL of MAb in the corresponding coating buffer, previously selected. After washing, the channels were blocked with 2% Bovine Serum Albumin (BSA, Sigma #A9647), 3% skim milk (Difco™ Laboratory, #232100), 1% casein (Sigma, #C5890), or OptiBlock solution. The antigen standards (10^7^ CFU/mL of Ac, 10 µg/mL of WSMoV-NP, and 10 µg/mL of MYSV-NP) prepared in corresponding blocking buffers were added and incubated for 20 min, followed by another washing step. Blocking solutions containing no antigens were used as negative controls. Corresponding AP-linked detection PAb of 16 µg/mL were added and incubated for 10 min. The final wash step (30 µL, twice) proceeded Attophos addition (10 µL, 15 min). All steps were performed at RT.

#### Optimization of capture/detection antibody concentrations

Based on the coating and blocking buffer selection results, 11E5, 2D6, and 5E7 were prepared at 2.5, 5, and 10 µg/mL, respectively, in the corresponding coating buffers. Using results from the traditional ELISA as baselines, AP-MPC, and AP-MYSV were prepared in OptiBlock at 4, 8, and 16 µg/mL, respectively. Individual checkerboard titration experiments were performed for each pathogen. Namely, MAb of three concentrations was added to the 12 columns of the microfluidic cartridge, while the 6 rows were filled with three concentrations of the paired PAb. The protein standards were diluted in OptiBlock at the following concentrations: 10^7^ CFU/mL of Ac, 10 µg/mL of WSMoV-NP, and 10 µg/mL of MYSV-NP. OptiBlock containing no antigens was included as negative control.

#### Verification of assay specificity

Each antibody pair was tested against various types of plant bacteria and virus standard samples. Using the same assay protocol, *Acidovorax citrulli* squash type B or SQB Ac (10^7^ CFU/mL), Fluorescent *Pseudomonas* spp. or Pf (10^7^ CFU/mL), *Delftia acidovorans* or D Ac (formerly known as *Commamonas acidovorans*, 10^7^ CFU/mL), and nucleocapsid protein of tomato yellow leaf curl virus or TYLCV (10 µg/mL) were tested together with Ac (10^7^ CFU/mL), WSMoV-NP (10 µg/mL) and MYSV-NP (10 µg/mL).

#### Determination of assay dynamic range

Following the same assay protocol, optimized concentrations of antibodies (10 µg/mL for 11E5, 2D6, and 5E7; 8 µg/mL for AP-MPC and AP-MYSV6) were used to determine assay sensitivity. In order to enhance assay sensitivity, a repetitive reagent-loading scheme was employed using the Ac panel as a model. Briefly, following the original 20-min antigen incubation, an extra 5-µL sample was added every 5 min in 10 and 20 increments, respectively. The rest of the protocol remained the same. To determine the sensitivity of the assays, different concentrations of Ac (0–10^8^ CFU/mL), WSMoV-NP (0-20 µg/mL), and MYSV-NP (0–20 µg/mL) standards in OptiBlock solution were tested in parallel with spiked plant extract containing the same pathogen concentrations. Assay sensitivity was experimentally determined based on the signals being greater than twice of the corresponding background values [32].

## Results and Discussion

### Traditional ELISA

The goal of repeating traditional ELISA assays in this study was to pre-optimize baseline parameters before the assay transfer, and to compare assay sensitivities between the two formats. This repeated experiment is crucial because optimal antibody concentrations, even for the same assay, may vary due to batch-to-batch variations in antibody production. Assay protocol as well as the choices of coating, blocking and washing buffers selection were based on our prior ELISA studies [28], [29]. Optimal concentrations of MAb were also taken from the previous study, in which the same batches of MAb were used [28]. Repetitive optimization with the same batch of MAb was avoided to ensure adequate supply of the antibodies for subsequent experiments in this study. Overall, we tested four different concentrations of PAb, and found that 4 µg/mL concentration yielded the highest S/N ratio for *Acidovorax avenae* subsp. *citrulli* (Ac), watermelon silver mottle virus (WSMoV) and melon yellow spot virus (MYSV) detection (Figure S1). Higher PAb concentrations above 4 µg/mL were not tested because further increasing concentrations of PAb, though may help increase the signals, contributed to significant rises in the background (OD absorbance reading >0.15, the maximal acceptable value). Based on data from the literature [28] and our experiments, 2.5 µg/mL of 11E5, 2D6, and 5E7, and 4 µg/mL of AP-MPC and AP-MYSV served as our starting point for assay transfer.

### Assay transfer and optimization

The microfluidic cartridge comprises an array of wells; each well is connected to a microfluidic spiral channel that serves as an individual reaction chamber [27]. To assemble the platform, the absorbent pad was placed on top of the cartridge holder and the microfluidic cartridge was snapped on top, sandwiching the absorbent pad as shown in Figure S2. The microchannel operates on the principle of capillary force that mediates transport of reagents into the chamber and facilitates sample incubation [27]. To operate, the reagent was simply added into the well by angling the pipette tip against the wall of the well to avoid introducing air bubbles that can block the reagent from passing through. The filled microchamber is automatically sealed and sandwiched between an exit of the microchannel and an underlying absorbent pad. Excess reagent is pushed out to drain onto the absorbent pad, and a subsequent step is accomplished by adding fresh reagent to replace an existing one in the channel.

#### Coating buffer selection

**Figure 2:**
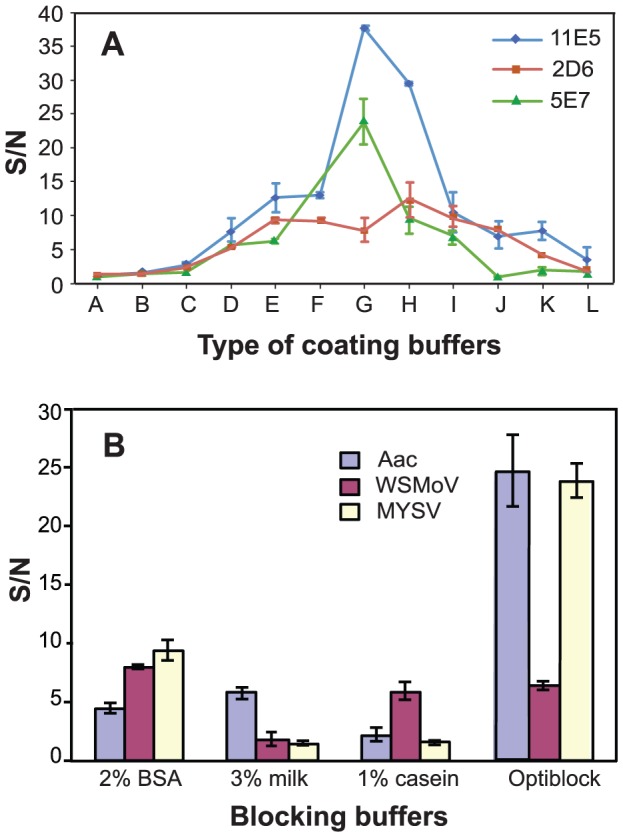
Coating and blocking buffer selection. A. Different coating buffers (Optibind A-L) were tested for maximum surface binding of 11E5, 2D6, and 5E7 (n = 3). B. Blocking buffers (2% BSA, 3% skim milk, 1% casein, and Optiblock solution) were tested for Ac, WSMoV, and MYSV detection (n = 3). An ideal blocking buffer resulted in highest S/N ratios. Error bars indicate ± standard deviations.

Selecting an appropriate coating buffer for each system was the first critical step to ensure successful assay transfer. Because relative binding performance among different coating buffers was evaluated, efficiency of MAb immobilization was detected through bound AP-linked anti-mouse IgG, bypassing the antigen and secondary binding steps. OptiBind A-L (proprietary contents) with pH values ranging from 3 to 8 were tested. Figure 2A reveals the best binding buffer for 11E5, 2D6, and 5E7 to be OptiBind G (pH 5.5), H (pH 6.5), and G, respectively. In a different study, sodium carbonate-bicarbonate buffer (pH 9.6)−commonly used coating buffer in ELISA assays−was also tested together with OptiBind A-L. Surprisingly, the buffer was found to exhibit the least binding capacity (data not shown). It is unclear as to why the ideal pHs for binding the same set of MAb are different between our traditional 96-well plate and the microfluidic microplate, given that both plates are made of polystyrene (according to the plate manufactures). One can stipulate on the proprietary coating of the microfludic plates.

#### Blocking buffer selection

To ensure that any slight discrepancies among different blocking buffers can be observed, high concentrations of antibodies (10 µg/mL for MAb, and 16 µg/mL for PAb) and antigens (10^7^ CFU/mL of Ac, 10 µg/mL of WSMoV-NP, and 10 µg/mL of MYSV-NP) were used. Four blocking buffers (2% BSA, 3% skim milk, 1% casein, and OptiBlock solution) were evaluated based on their ability to suppress background from non-specific binding. Results in Figure 2B show that OptiBlock yields highest S/N ratio for Ac and MYSV. The solution gave 4–10 times higher signal and 2–4 times lower background than skim milk and casein. Even though BSA gave 8–9 times higher signal than OptiBlock for WSMoV, the solution caused considerably high background (∼7 times higher) as shown in Figure S3. This could result in low assay sensitivity and compromised assay specificity, therefore OptiBlock was selected for all pathogen detection panels in further experiments.

#### Optimization of capture and detection antibody concentrations

**Figure 3:**
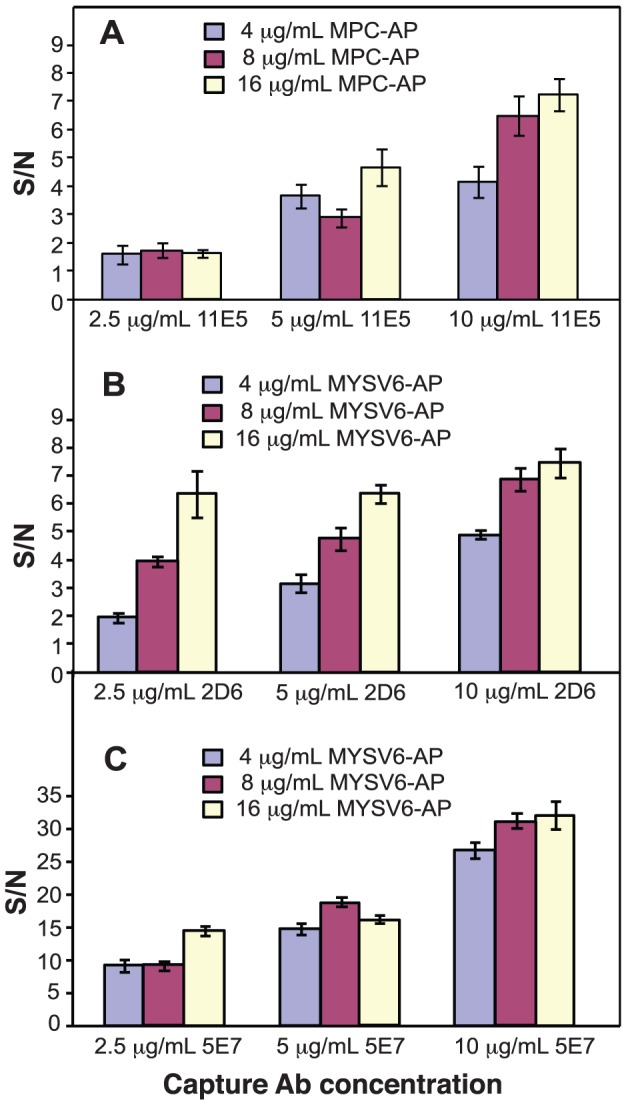
Optimization of antibody concentration. Nine different conditions for each disease panel were tested on the microfluidic platform using combinations of three concentrations of capture Ab (11E5, 2D6, and 5E7) and three concentrations of detection Ab (MPC-AP, MYSV6-AP). Panel A–C show results for Ac, WSMoV, and MYSV detection, respectively (n = 4). The S/N ratios were plotted for each of the conditions tested. Error bars indicate ± standard deviations.

Following the manufacturer's guideline of using 1-, 2-, and 4-fold standard Ab concentrations, combinations of 2.5, 5, and 10 µg/mL MAb (11E5, 2D6, and 5E7) and 4, 8, and 16 µg/mL AP-linked PAb (AP-MPC, and AP-MYSV) were tested using a checkerboard approach. Results in Figure 3 indicate that the signal strength was largely governed by the concentration of MAb, and therefore the highest concentration of 10 µg/mL was selected for all targets. Although it was technically feasible to further increase the concentration of MAb beyond 4× standard concentration, doing so would require an even greater amount of MAb, driving up the overall assay cost unnecessarily. At 10 µg/mL MAb, it was clearly observed that the differences in the S/N ratios between 8 and 16 µg/mL of PAb was gradual (∼8–11% increase) compared to the much more pronounced distinctions (∼40–57%) between 4 and 8 µg/mL, especially in the Ac and WSMoV panels. The effect of PAb concentrations was less dramatic for MYSV panel, which showed overall 4–5 times higher S/N than Ac and WSMoV panels across all MAb concentration ranges. Based on this data (n = 4), 10 µg/mL of 11E5, 2D6, and 5E7, and 8 µg/mL of AP-MPC and AP-MYSV were selected for Ac, WSMoV, and MYSV detection, respectively.

These optimization results indicate that the microfluidic system requires higher concentrations of capture and detection antibodies than the traditional plate format. This is partly due to the 50× higher surface to volume ratio [27], [33], which leads to a lower effective concentration on the reactive surface. Other factors governing mass transport properties such as diffusivity, flow rate, and channel geometry also contribute to different optimal concentration ranges and reaction kinetics [27]. Despite higher antibody concentrations required (4× and 2× for MAb and PAb, accordingly), the microfluidic system still saves 5–10 times overall reagent consumption due to its 20 times lower volume requirement (100 µL vs. 5 µL per reaction).

### Assay specificity and sensitivity

#### Verification of assay specificity

**Figure 4:**
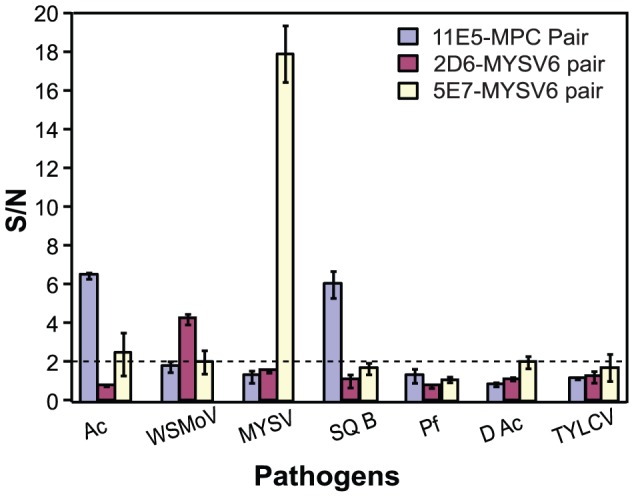
Specificity determination. Specificity of the 11E5/MPC, 2D6/MYSV6, and 5E7/MYSV6 antibody pairs was tested for Ac, WSMoV, and MYSV, respectively. The antibody pairs were tested against bacteria (Ac, SQB, Pf, and DAc) and viral (TYLCV) protein standards (n = 3). The data shows averaged S/N values with error bars representing standard deviations. The dotted horizontal line indicates the threshold (or the cutoff value) of S/N = 2 (twice of values obtained from negative controls).

To confirm integrity and stringency of the antibodies in the microfluidic environment, assay specificity tests were performed. The antibody pairs (11E5/MPC, 2D6/MYSV6, and 5E7/MYSV6) were tested against bacteria samples: *Acidovorax avenae* subsp. *citrulli* (Ac), *Acidovorax citrulli* squash type B (SQB), Fluorescent *Pseudomonas* spp. (Pf), and *Delftia acidovorans* (DAc, formerly known as *Commamonas acidovorans*); and viral protein standards: watermelon silver mottle virus (WSMoV), melon yellow spot virus (MYSV), and tomato yellow leaf curl virus (TYLCV). High concentration protein standards of 10^7^ CFU/mL for bacteria and 10 µg/mL for viruses were used so that any ambiguities due to low sensitivity are minimized. Using a S/N = 2 threshold, Figure 4 shows the 11E5/MPC pair exhibiting selectivity towards Ac and SQB, and against Pf, D Ac, and TYLCV as expected according to highly specific characteristics of 11E5 MAb previously reported [29]. For Tospoviruses, the 2D6/MYSV6 and 5E7/MYSV6 Ab pairs were selected specifically for WSMoV and MYSV, respectively [34]. The results in Figure 4 did not exhibit any cross reactivity of 2D6/MYSV6 with bacteria or Geminivirus TYLCV, demonstrating that antibody specificity was not compromised in the microchannel environment. With an exception of a slight reactivity towards Ac when using S/N = 2 as a cutoff, the 5E7/MYSV6 pair specifically detected MYSV with the highest S/N ratio for true positives. Given such a wide distinction between positive and negative results (9× difference), a higher threshold level (i.e. S/N>3) presents a more appropriate cut-off value for this assay. These results, nevertheless, confirmed that our detection panels remained highly specific towards Ac, WSMoV, and MYSV.

#### Assay Sensitivity

After successful assay transfer and optimization, the final fine-tuning to achieve maximum sensitivity involved repetitive sample loading. Since there is no sample/reagent removal between steps, more samples can be easily loaded without changing the existing protocol. In this study, increments of 5-µL sample were added after the 20-min sample incubation. The incubation time for these additional steps was reduced to 5 min to keep the overall assay time relatively short. The 5-min incubation is, however, long enough to accommodate any flow variations among different microchannels and to allow for >90% adsorption [27]. Due to the “fully developed region” operation regime of pressure-driven flow that is a unique characteristic of a microchannel structure [35], higher portion of bound molecules can be achieved in this format than those in its traditional 96-well counterpart.

**Figure 5:**
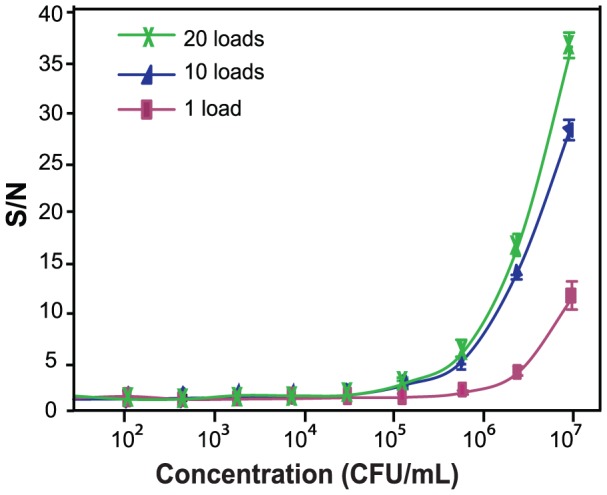
Study of repetitive sample loading. Effect of repetitive sample loading on assay dynamic range was investigated, using Ac as a model. The plot indicates that multiple loading helps increase assay sensitivity (n = 3). Error bars indicate ± standard deviations.

Using Ac as a model, Figure 5 compares effects of 1, 10, and 20 sample loads on assay sensitivity across the concentration range of 0–10^8^ CFU/mL (The 0 CFU/mL was omitted in the graph due to the log-scale plotting, and the 10^8^ CFU/mL was excluded due to signal overflow). At the concentrations above S/N = 2 thresholds, extra loading contributed to enhanced assay sensitivity, especially during the first 10 loads. An increase in sensitivity became less prominent as the number of loads increased from 10 to 20 (*i.e.* limits of detection or LOD for 1, 10, and 20 loads are 2–6×10^6^, 4×10^5^, and 1–4×10^5^ CFU/mL, respectively). In addition, the 20-load protocol doubled the assay time (from the original 105 min to 200 min). For these reasons, the 10-repetition loading scheme was selected for further assay sensitivity determination and real sample testing. Although this approach does require a greater amount of sample, it does not pose a great deal of concern to end-users because the plant samples are usually abundant. In some cases, as shown here, assay sensitivity can be improved by an order of magnitude via this loading technique.

**Figure 6:**
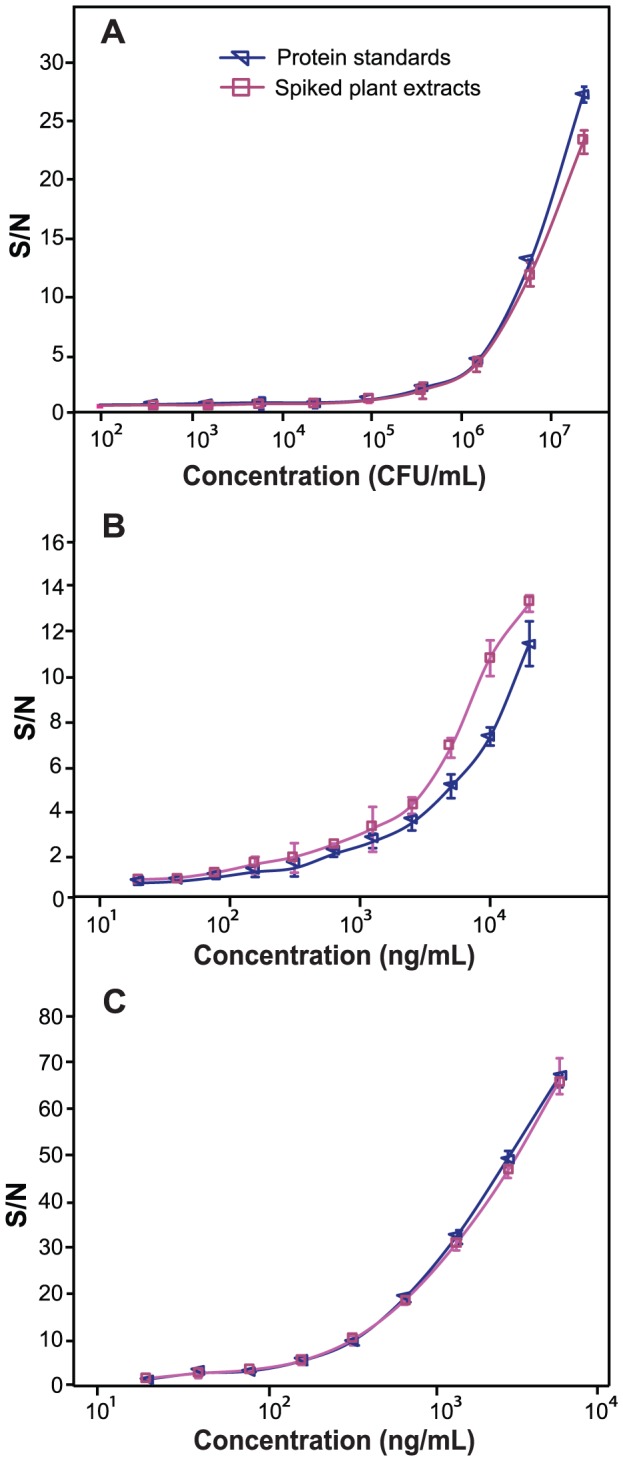
Sensitivity determination of the microfluidic platform. Comparison of assay dynamic range for Ac (A), WSMoV (B), and MYSV (C) detection between protein standards and spiked plant extracts (n = 3) by the microfluidic platform. Error bars indicate ± standard deviations.

Detection dynamic ranges for each pathogen on the microfluidic system were shown in Figure 6. Routinely practiced for ELISA sensitivity determination, the S/N = 2 was used as a threshold [28], [32]. An exception of S/N = 3.5 was chosen for MYSV due to the results from our specificity test. Limits of detection (LODs) for Ac, WSMoV, and MYSV were 4×10^5^ CFU/mL, 625 ng/mL, and 80 ng/mL; compared to the traditional ELISA method with assay sensitivities of ∼5×10^6^ CFU/mL, 1.25 µg/mL, and 300 ng/mL for Ac, WSMoV, and MYSV, respectively (Figure S4). In other words, the microfluidic-based technology offers 12.5-, 2-, and 4-fold enhanced sensitivity for Ac, WSMoV, and MYSV, respectively. These levels of sensitivity are on par with, if not greater than, those obtained from the microsphere immunoassay in the previously published work by our group. According to the literature, the microsphere method offers 7-, 3-, and 4-fold enhanced sensitivity for Ac, WSMoV and MYSV, respectively [28].

Given that WSMoV and MYSV are members of the *Bunyaviridae* family, which generally has 2,100 copies of NP per viral particle [36] with each NP weighing ∼31 kDa [37], assay sensitivity can also be calculated in terms of viral particle copy numbers. Our studies indicated that the microfluidic platform was sensitive down to 6×10^9^ WSMoV particles/mL, and 8×10^8^ MYSV particles/mL, respectively. Pearson's correlation coefficients between protein standards and spiked plant extracted (for both assay formats) are 0.989–0.999, indicating highly robust systems. Larger variations between protein standard and spiked plant extracts were only observed in nucleocapsid proteins (NPs) at high concentrations, and not cell lysates. This can be explained by our observation that stored NP protein suspensions are prone to form particulates that are sometimes difficult to break even after vigorous sonication and vortexing. Nevertheless, this variation did not seem to affect the LOD.

### Real samples testing

We evaluated technological feasibility and practical performance of the microfluidic technology in detecting seedborne pathogens by testing natural watermelon leaves. To avoid biases due to different sample concentrations, a stock supernatant from each plant sample was diluted using the same dilution factor for both platforms. The samples were also immediately tested in parallel to prevent potential variations due to time-dependent sample degradation. Table 2 summarizes results obtained from 19 samples that were previously tested, upon collection, for Ac by ELISA and microsphere immonuassay [28], and for tospovirus (WSMoV and MYSV) by PTA-ELISA together with reverse transcription PCR [29], [30].

**Table 2:** Comparison of *Acidovorax avenae* subsp. *Citrulli* (Ac), watermelon silver mottle virus (WSMoV), and melon yellow spot virus (MYSV) detection in real watermelon leaf samples by the microfluidic vs. traditional ELISA (n = 3).

	Microfluidic method			Traditional method			
Sample #	Ac	WSMoV	MYSV	Ac	WSMoV	MYSV	Infection type*
1	+	−	−	+	−	−	Ac
2	+	−	−	+	−	−	Ac
3	+	−	−	+	−	−	Ac
4	+	−	−	+	−	−	Ac
5	+	−	−	+	−	−	Ac
6	−	+	−	−	+	−	CaCv#
7	−	+	−	−	+	−	WSMoV
8	−	+	−	−	−	−	WSMoV
9	−	−	−	−	−	−	WSMoV
10	−	−	−	−	−	−	WSMoV
11	−	−	+	−	−	+	MYSV
12	−	−	+	−	−	+	MYSV
13	−	−	+	−	−	+	MYSV
14	−	−	+	−	−	+	MYSV
15	−	−	+	−	−	+	MYSV
16	−	−	−	−	−	−	H-watermelon
17	−	−	−	−	−	−	H-watermelon
18	−	−	−	−	−	−	H-cucumber
19	−	−	−	−	−	−	H-pumpkin

* Infection type was previously identified by ELISA and microsphere immunoassay for Ac, and by PTA-ELISA and RT-PCR for WSMoV and MYSV.

# CaCv denotes capsicum chlorosis virus. CaCV is categorized under WSMoV serogroup, and thus can be detected by antibodies specific to WSMoV [34].

“H” denotes healthy.

Detection results from the traditional and the microfluidic-based ELISA formats were in an excellent agreement with each other, and also in agreement with the predetermined results (indicated as “infection type” in table 2) for all Ac- and MYSV-infected samples. Two out of five samples previously determined as infected with tospovirus WSMoV serogroup (WSMoV and CaCV) were detected positive by both ELISA methods, while one sample was detected positive only by the microfluidic technique. This could result from the higher sensitivity offered by the microfluidic system, as shown earlier. However, two out of five samples determined as WSMoV-positive by the PTA-ELISA yielded negative results by both sandwich platforms. This suggested that the sandwich ELISA might not be as suitable for detecting WSMoV as the PTA-ELISA. It is also possible that the extraction buffer used in the PTA-ELISA containing an anti-oxidation agent helped suppress activities of phenolics released by some grounded plants from inhibiting the immunoassay [38]. The healthy samples were confirmed negative by both platforms. Overall, this proof-of-concept study has demonstrated the first implementation of microfluidic-based ELISA as a superior alternative diagnostic method for plant pathogen detection. Test results could be obtained within 90–140 min less time (not including the overnight incubation needed for traditional ELISA), consuming 2–20 folds less sample and 5–10 folds less antibody. This totals to an overall 65% or $95 cost savings/plate (Table S1).

## Conclusions

In this paper, we present a new and simpler approach to perform sandwich ELISA for rapid and accurate screening of seedborne pathogens using microfluidic technology. Taking advantages offered by the microchannel structure, the micro-scale reaction volume helps minimize sample consumption, while the enhanced reaction kinetics cuts down the assay time by more than half, resulting in net cost savings of 65%. As a proof-of-concept study, we have shown that the microfluidic-based ELISA can detect Ac, WSMoV, and MYSV at 12.5, 2 and 4 times greater sensitivity than the traditional ELISA method, respectively. The optimized diagnostic systems were also demonstrated in real plant samples. Equipped with superior assay performance, preferable cost structure, and flexibility for robotic integration, this verified system provides the first step toward a new benchmark for fully automated and large-scale quality control in agricultural products.

## Supporting Information

Figure S1 **Optimization of PAb concentrations** Different concentrations for MPC-AP and MYSV6-AP were tested for Ac, WSMoV, and MYSV detection in traditional ELISA (n = 3). Error bars indicate ± standard deviations.
(EPS)

Figure S2 **Optical micrograph of the microfluidic ELISA platform** A. The apparatus includes a microplate holder, an absorbent pad, and a microfluidic cartridge. B. The assembled platform ready for operation.
(EPS)

Figure S3 **Effect of blocking buffers on the background readout** Background levels for Ac, WSMoV, and MYSV panels were obtained from negative control experiments using 2% BSA, 3% skim milk, 1% casein, and OptiBlock as blocking solutions (n = 3). Error bars indicate ± standard deviations.
(EPS)

Figure S4 **Sensitivity determination of the traditional ELISA format** Comparison of assay dynamic range for Ac, WSMoV, and MYSV detection between protein standards and spiked plant extracts (n = 3) by the traditional ELISA method. Error bars indicate ± standard deviations.
(EPS)

Table S1 **Assay costs** Comparison of cost breakdowns between the traditional and microfluidic-based ELISA platforms.
(DOCX)
