# Supplementary material for: Implementation of Microfluidic Sandwich ELISA for Superior Detection of Plant Pathogens
Source: PLoS One. 2013 Dec 23;8(12):e83231. doi: 10.1371/journal.pone.0083231 (PMC3871650; doi:10.1371/journal.pone.0083231)
Supplement: Table S1 — Assay costs Comparison of cost breakdowns between the traditional and microfluidic-based ELISA platforms. (DOCX) [file pone.0083231.s005.docx]

**Table S5**

| **Items** | **Microfluidic** | **Traditional** |
| --- | --- | --- |
| Buffers/reagents | $13.50/plate | $30/plate |
| Samples | 5-50 μL | 100 μL |
| Antibodies | $8/plate | $75/plate |
| Plates | $14.50/plate | $3.70/plate |
| Labor *($10/hr wage*) | 105 min/plate | 240 min/plate |
| *Total* | *$53.50/test* | *$148.70/test* |

Note: Price quotes are calculated before taxes.
